# Diastolic ventricular function in persistent pulmonary hypertension of the newborn

**DOI:** 10.3389/fped.2023.1175178

**Published:** 2023-06-26

**Authors:** Kévin Le Duc, Thameur Rakza, Jean Benoit Baudelet, Mohamed Riadh Boukhris, Sébastien Mur, Ali Houeijeh, Laurent Storme

**Affiliations:** ^1^ Department of Neonatology, Pôle Femme-Mère-Nouveau-Né, Hôpital Jeanne de Flandre, Centre Hospitalier Universitaire de Lille, Lille, France; ^2^ University of Lille, CHU Lille, ULR 2694—METRICS: Évaluation des Technologies de Santé et des Pratiques Médicales, axe Environnement Périnatal et Santé, Lille, France; ^3^ Center for Rare Disease Congenital Diaphragmatic Hernia, Jeanne de Flandre Hospital, Centre Hospitalier Universitaire de Lille, Lille, France; ^4^ Department of Pediatric Cardiology, Jeanne de Flandre Hospital, Centre Hospitalier Universitaire de Lille, Lille, France

**Keywords:** PPHN, newborn, right ventricular performance, diastolic dysfunction, tissue doppler imaging

## Abstract

**Background:**

Persistent pulmonary hypertension of the newborn (PPHN) is usually considered a consequence of impaired pulmonary circulation. However, little is known regarding the role of cardiac dysfunction in PPHN. In this study, we hypothesized that the tolerance for pulmonary hypertension in newborn infants depends on the biventricular function. The aim of this study is to evaluate biventricular cardiac performance by using Tissue Doppler Imaging (TDI) in an healthy newborn infants with asymptomatic pulmonary hypertension and in newborn infants with PPHN.

**Methods:**

Right and left cardiac function were investigated using conventional imaging and TDI in 10 newborn infants with PPHN (“PPHN”) and 10 asymptomatic healthy newborn infants (“asymptomatic PH”).

**Results:**

Systolic pulmonary artery pressure (PAP) as assessed by TDI and the mean systolic velocity of the right ventricular (RV) free wall were similar in both groups. The isovolumic relaxation time of the right ventricle at the tricuspid annulus was significantly longer in the “PPHN” than in the “asymptomatic PH” group (53 ± 14 ms vs. 14 ± 4 ms, respectively; *p* < 0.05). Left ventricular (LV) function was normal in both groups with a systolic velocity (S'LV) at the LV free wall groups (6 ± 0.5 cm/s vs. 8.3 ± 5.7 cm/s, *p* > 0.05).

**Conclusion:**

The present results suggest that high PAP with or without respiratory failure is not associated with altered right systolic ventricular function and does not affect LV function in newborn infants. PPHN is characterized by a marked right diastolic ventricular dysfunction. These data suggest that the hypoxic respiratory failure in PPHN results, at least in part, from diastolic RV dysfunction and right to left shunting across the foramen ovale. We propose that the severity of the respiratory failure is more related to the RV diastolic dysfunction than the pulmonary artery pressure.

## 1. Introduction

Persistent pulmonary hypertension of the newborn (PPHN) is a clinical syndrome associated with diverse neonatal cardiopulmonary diseases, such as sepsis, meconium aspiration, and respiratory distress syndrome, but it can also be idiopathic (1). Physiologically, PPHN is characterized by the failure of the pulmonary circulation to dilate at birth. This leads to sustained elevation of PVR, causing extrapulmonary right-to-left shunting of blood across the DA and foramen ovale and severe hypoxemia (1). PPHN contributes significantly to high morbidity and mortality in hypoxemic newborns, but its pathophysiology remains poorly understood.

Most of the studies regarding PPHN pathogenesis have focused on the primary role of pulmonary circulation (1). Abnormal pulmonary vasoreactivity and structural changes in pulmonary vessel walls have been considered the main causes of PPHN (2). However, there is some evidence that cardiac dysfunction may play a major role in PPHN (3, 4). Severe pulmonary hypertension increases RV afterload, which may result in RV failure. The resulting elevation of RV telediastolic pressure causes right-to-left shunting of blood through the foramen ovale and worsens hypoxemia. In PPHN, hypoxemia may impact the quality of coronary perfusion, and LV functions might subsequently be impaired (5). Moreover, decreased pulmonary blood flow and venous return to the left atrium may decrease left atrial (LA) pressure and contribute to right-to-left shunting through patent foramen ovale, worsening hypoxemia. In addition, LV diastolic dysfunction can increase LA pressure producing pulmonary venous hypertension, pulmonary congestion with or without hemorrhage, and decreased pulmonary compliance.

Due to a high degree of interdependence between the right and left ventricles owing to the presence of common structures (the interventricular septum and the inextensible pericardium), changes in right ventricle size and geometry may alter LV function (6, 7). This may explain why PPHN is usually associated with low systemic pressure and cardiac output requiring cardiac support (1). However, the relative roles of abnormal pulmonary circulation and cardiac dysfunction in PPHN remain unknown.

Tissue Doppler Imaging (TDI) is used to evaluate the right cardiac performance. Myocardial TDI is a tool which allows a quantitative evaluation of the cardiac wall's movements by measurement of the intramyocardic velocities in real time. Compared to conventional Doppler techniques, TDI is an important advance in the assessment of cardiac function given its relative pre- and post-load independence and reproducibility (8). Whereas conventional Doppler techniques focus on the low intensity and high velocity echoes of blood flow, TDI measures the high intensity, low velocity echoes of the myocardium. The physical differences between the signal returning from moving blood and cardiac tissue motion is that the velocity in the hydraulic part (blood) is higher than in tissue (mechanic part) (9). TDI has been established as a reliable echocardiographic tool to assess ventricular dysfunction in children with pulmonary hypertension (10–12).

We hypothesize that the tolerance of pulmonary hypertension in newborn infants is dependent on cardiac function. In order to investigate this hypothesis, we conducted a comparative analysis of cardiac function between newborn infants with asymptomatic pulmonary hypertension and those with pulmonary hypertension associated with severe respiratory failure.

## 2. Materials and methods

### 2.1. Population

This prospective observational study was conducted at the Nursery and Neonatal Intensive Care Units of Lille University Hospital, France. We included newborn infants with a gestational age >36 weeks and PPHN (group “PPHN”) as defined by severe respiratory failure [mechanical ventilated, FiO2 >35%, and inhaled nitric oxide (iNO) treatment]. Asymptomatic newly born infants with no respiratory failure were included in the “asymptomatic PH” group. In both groups, newborn infants had a postnatal age of <2 h. The exclusion criteria were congenital cardiopathy, gestational age <36 weeks, use of inotropic drugs, and hypovolemia. Informed consent was obtained from the parents. This study was approved by the National Commission on Informatics and Liberty.

### 2.2. Method

All examination procedures were performed to provide a comprehensive echocardiographic examination for assessing RV and LV systolic and diastolic function using several indexes. Tissue Doppler Imaging (TDI) was performed in the supine position using an ultrasound machine (GE Vivid 7; GE Medical Systems, Princeton, NJ, USA). One investigator performed all the TDI procedures. We used TDI to evaluate right cardiac performance. Myocardial TDI is a tool that allows the quantitative evaluation of cardiac wall movements via measurement of intramyocardial velocities in real time. Whereas conventional Doppler techniques focus on the low-intensity, high-velocity echoes of blood flow, TDI measures the high-intensity, low-velocity echoes of the myocardium. There is a physical difference between the signal returning from moving blood and cardiac tissue motion because the velocity in the hydraulic part (blood) is higher than that in the mechanic part (tissue) (9). TDI has been established as a reliable echocardiographic tool to assess ventricular dysfunction in children with pulmonary hypertension (11, 13, 14). To optimize the performance of TDI in our neonatal population, each newborn having an echocardiogram by an advanced cardiologist is cocooned by a neonatal nurse or one of the parents to decrease anxiety and agitation of the patient. Before ultrasound is started, nonpharmacological approaches like non-nutritive sucking or sucrose is used to manage neonatal pain and stress avoiding major tachycardia (15). To optimize the quality of our TDI measurements, we respected an angle of insonation less than 20° to avoid underestimating velocities, a velocity range ± 0.16 m/s to avoid aliasing and a sample area of 2–3 mm (10).

Doppler measurements represent the average of three beats. Special care was taken to prevent angulation between the ultrasound beam and blood flow. The following Doppler parameters (pulsed, color, and tissue) were measured:

– Left ventricular shortening fraction (LVSF) from a parasternal long-axis view (16).
– Internal diameter and maximal velocity across the DA by color and pulsed Doppler from the high left parasternal view (14). PAP was evaluated using right-to-left maximal blood flow velocity and the Bernoulli formula (|*Δ*P = 4V^2^|), which is a noninvasive method supported by the literature (17, 18).
– Mitral and tricuspid inflow velocities using the apical window with a pulsed-wave Doppler sample volume length of 2 mm placed at the mitral and tricuspid valve tips. Tricuspid and mitral inflows were assessed for peak early diastolic velocity (E) and peak late diastolic velocity (A).
– Systolic velocity (S'), early diastolic velocity (E'), and late diastolic velocity (A') by TDI at the RV free wall (lateral area of the tricuspid annulus on the apical view) and at the LV free wall (lateral area of the mitral annulus on the apical view) by TDI pulsed spectral imaging of a sample volume length of 2–5 mm is placed within the myocardial wall of interest, interrogating the wall motion parallel to the cursor orientation. Images of the right and left ventricles were chosen to minimize the angle of incidence between the scanlines and the motion of the base of the heart. Filters were set to exclude high-frequency signals, and gains were set to obtain clear tissue signals with minimal background noise.
– Isovolumic relaxation time of the right ventricle (IVRT; the time between the end of the S' wave and the beginning of the E' wave). The IVRT is the time between the closure of the pulmonary valve and the opening of the tricuspid valve. During this phase, the ventricle is a closed cavity whose volume does not change while the pressure generated during the systole decreases exponentially.
– Inferior vena cava diameter (mm) using the low subcostal view;
– To calculate the E/E' ratio, off-line analyses included measurements of E and E' for both ventricles.

### 2.3. Statistical analysis

The results are presented as means ± standard error of the mean. The data were analyzed using factorial analysis of variance. Intergroup differences were analyzed using the Kruskal–Wallis test (StatView for PC; Abacus Concepts, Berkeley, CA, USA). The Mann–Whitney test (independent values) and paired Wilcoxon rank test (paired values) were used to compare groups. *P* values < 0.05 were considered statistically significant.

## 3. Results

Twenty newborn infants were included in the study (10 in each group). The mean gestational age was 39 ± 1 weeks in the “asymptomatic PH” group and 38 ± 2 weeks in the “PPHN” group (p = 0.6). The mean birth weights were similar in each group (“asymptomatic PH” group: 3,400 ± 500 g; “PPHN” group: 3,000 ± 600 g; *p* = 0.08). PPHN-related diseases are shown in Table 1. In the “PPHN” group, the echocardiographic assessment was performed at a median age of 18 h [12–46]. In the “asymptomatic PH” and “PPHN” groups, the shunt through the DA was bidirectional in every infant. The mean pressure gradients across the DA were 0.7 ± 1.0 mmHg and 1.2 ± 2.0 mmHg in the “asymptomatic PH” and “PPHN” groups, respectively (*p* = 0.64). The mean DA diameter was equal between the two groups (3.7 ± 0.7 mm vs. 2.2 ± 1.6 mm, respectively; *p* = 0.09).

**Table 1:** Causes of the respiratory failure in the group “PPHN”.

	*N*
Hyaline membrane disease	3
Congenital diaphragmatic hernia	2
Meconium aspiration syndrome	2
Early onset sepsis	2
Idiopathic	1

### 3.1. Right ventricular function

**Figure 1:**
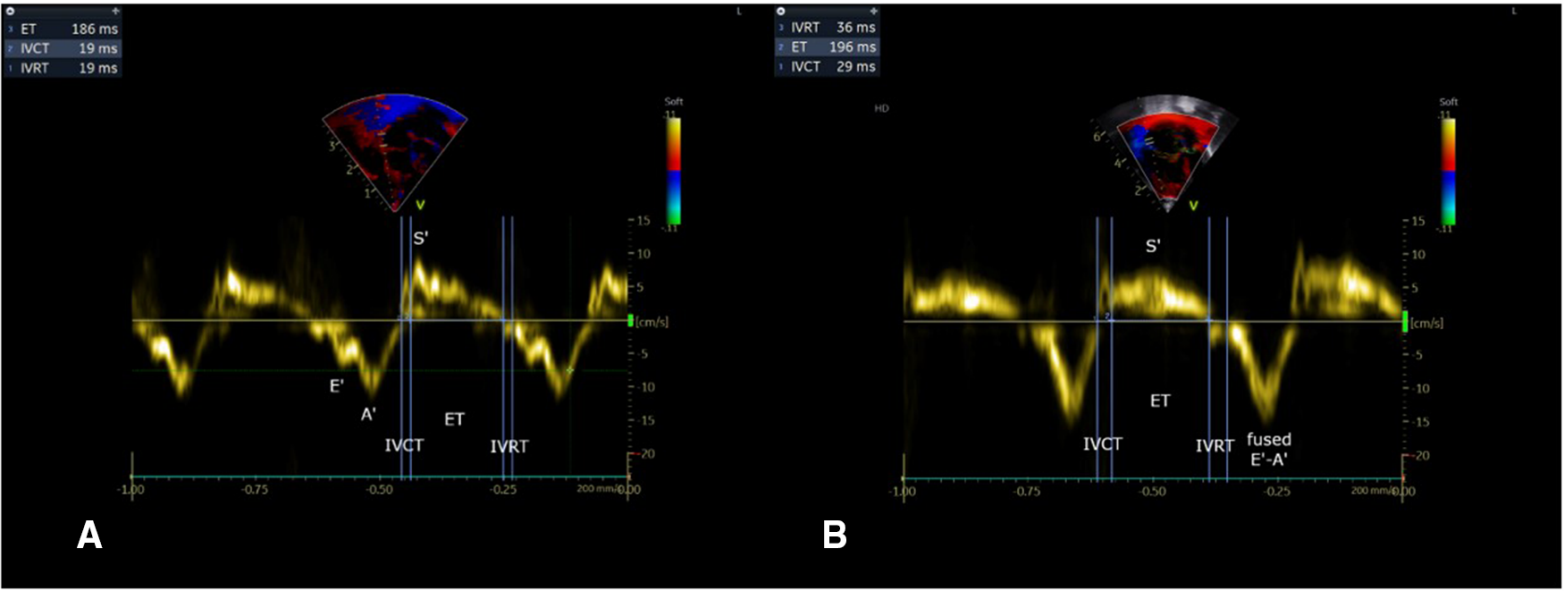
Pulsed-wave o s s ler imaging at the lateral tricuspid annulus with measurements of cardiac cycle time intervals used for IVRT. (**A**)/As rn s tomatic PH group. (**B**)/PPHN group. IVRT RV is ned in the PPHN group (*p *< 0.05).

**Figure 2:**
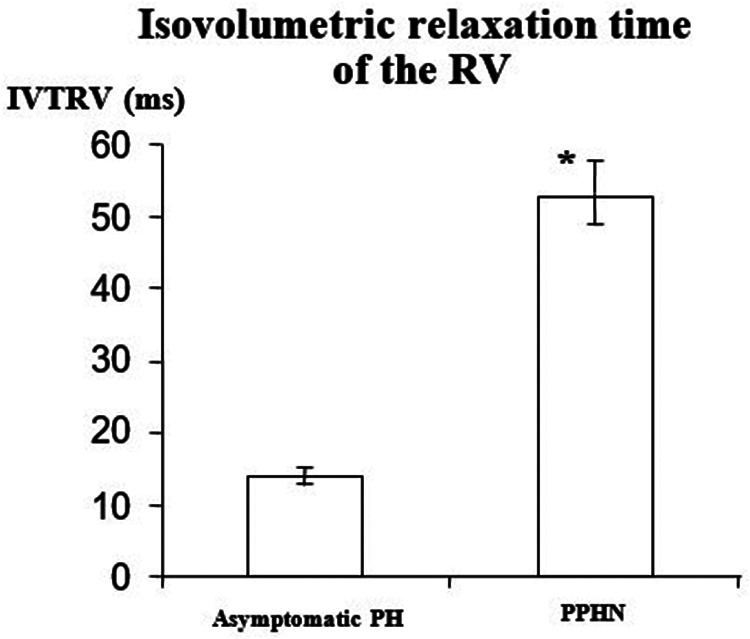
Right ventricular diastolic function evaluated by the measurement of the Isovolumic Relaxation Time of the Right Ventricle (IVRT) at the tricuspid annulus. IVRT was significantly longer in the group "PPHN" (*n *= 10) than in the group "asymptomatic pulmonary hypertension" (*n *= 10). Data are mean±SD: *, *p* < 0.005.

Figure 1 Illustrates the methodology for performing tissue Doppler imaging (TDI) on the right ventricle. The IVRT was significantly longer in the “PPHN” than in the “asymptomatic PH” group (53 ± 14 ms vs. 14 ± 4 ms, respectively; *p* < 0.05 Figure 2). All newborn infants in the “PPHN” group had a right-to-left or a bidirectional shunt across the foramen ovale. By contrast, the “asymptomatic PH” group had a left-to-right shunt across the foramen ovale (*p* < 0.05). No statistically significant difference in the mean diameters of the inferior vena cava was found between the two groups (Table 2). No difference in E wave velocity at the tricuspid annulus was found between the “PPHN” and “asymptomatic PH” groups (61 ± 21 cm/s vs. 63 ± 13 cm/s, respectively; *p* = 0.42). No significant difference in systolic velocity (S’RV), early diastolic velocity (E’RV), or late diastolic velocity (A’RV) was found by TDI at the RV free wall, and E/E’RV did not differ significantly between the two groups (Table 3).

**Table 2:** General parameters of the studied population.

Parameters	Asymptomatic PH	PPHN	*p*
Weight (g)	3,400 ± 500	3,000 ± 600	0.08
Gestational age (W)	39 ± 1	38 ± 2	0.6
Inferior vena cava diameter (mm)	5.3 ± 1.3	6.2 ± 2.2	0.8
DA diameter (mm)	3.7 ± 0.7	2.2 ± 1.6	0.09
DA mean pressure gradient (mmHg)	0.7 ± 1	1.2 ± 2	0.64
Heart rate (bpm)	139 ± 20	136 ± 21	0.34

Data are presented as mean ± SD.

DA, Ductus arteriosus; PA, Pulmonary Artery.

**Table 3:** Echographic parameters of the ventricular function.

	Parameters	Asymptomatic PH	PPHN	*p*
Left ventricular function	EM (cm/s)	80 ± 13	57 ± 18	0.07
	S’LV (cm/s)	6.0 ± 0.5	8.3 ± 5.7	0.12
	E’LV (cm/s)	6.6 ± 1.6	5.5 ± 2.1	0.39
	A’LV (cm/s)	3.8 ± 1.1	4.9 ± 3.4	0.71
	E/E“LV	10.3 ± 3.1	9.2 ± 2.9	0.39
	LVSF (%)	48.82 ± 11.68	50.19 ± 14.87	0.55
Right ventricular function	ET (cm/s)	63 ± 13	61 ± 21	0.42
	S’RV(cm/s)	7.2 ± 1.5	7.5 ± 1.7	0.47
	E’RV (cm/s)	10.4 ± 3.5	9.9 ± 4.0	0.82
	A’RV (cm/s)	4.7 ± 4.1	5.0 ± 3.5	0.34
	E/E‘RV	6.86 ± 3.01	6.67 ± 2.23	0.58
	IVRTRV (ms)	14 ± 4	53 ± 14	<0.05
Shunt across PFO (%)	Left-to-Right Bidirectional Right-to-Left	100 0 0	0 80 20	** **

Data are presented as mean ± SD.

LV, left ventricle; RV, right ventricle; EM, E wave at the mitral annulus; ET, E wave at the tricuspid annulus; Systolic velocity (S’LV, S’RV), early diastolic velocity (E’LV, E’RV)) and late diastolic velocity (A’LV, A’RV) measured by tissue Doppler at respectively the left and the right ventricles free walls.

IVRTRV, Isovolumic relaxation time of the right ventricle; LVSF, left ventricle shortening fraction; PFO Patent Foramen Ovale.

#### 3.2. Left ventricular function

Systolic velocity (S’LV) at the LV free wall and the LVSF were similar in the two groups (6 ± 0.5 cm/s vs. 8.3 ± 5.7 cm/s, *p* = 0.12) and (49 ± 12% vs. 50 ± 15%, *p* = 0.55) (Table 3). In addition, no significant difference in LV diastolic function was found between groups (E'LV 6.6 ± 1.6 m/s vs. 5.5 ± 2.1 cm/s, *p* = 0.39), EM (80 ± 13 cm/s vs. 57 ± 18 cm/s, 0.07), and E/E'LV (10.3 ± 3.1 vs. 9.2 ± 2.9, *p* = 0.39); Table 3).

## 4. Discussion

In this study, we investigated the role of cardiac function in pulmonary hypertension tolerance in newborn infants. We evaluated diastolic and systolic ventricular function using spectral TDI in newborn infants with pulmonary hypertension with and without associated severe respiratory failure. Whereas PAP was similar, the RV isovolumic relaxation time was longer in newborn infants with respiratory failure. These results suggest that respiratory failure in pulmonary hypertension of the newborn is associated with RV diastolic dysfunction.

Several previous series have studied ventricular function in patients with PPHN. RV dysfunction is a determinant of illness severity in infants with pulmonary hypertension (18). Previous studies have examined the role of ventricular function in persistent pulmonary hypertension of the newborn (19–21). However, the use of tissue Doppler imaging (TDI) in neonates is uncommon, and our study emphasizes the importance of this modality in evaluating cardiac function in newborns with PPHN*.*Inadequate visualization of the RV free wall precludes accurate measurements of changes in the RV fractional area and ejection fraction (22). Spectral TDI at the tricuspid annulus allows a quantitative evaluation of ventricle wall movements (8). Systolic TDI velocity at the right ventricle free wall (S'RV) quantifies the velocity of the tricuspid annulus displacement toward the apex. It is considered a preload- and afterload-independent marker of RV systolic function (22, 23). Our systolic S velocity and diastolic E' and A' velocities in infants without respiratory failure agree with previous data (24). Due to the rapid diastolic filling of the right ventricle caused by the high heart rate, the tissue velocity signals can overlap or fuse together, making it visually challenging to distinguish the individual E' and A' waves. In order to mitigate the fusion effect, our cardiologists implemented a waiting period after sucrose administration and cocooning to allow the heart rate to decrease (25). Reduced systolic myocardial velocities (S') were found in newborn infants over 15 days of age with pulmonary hypertension, which suggests impaired systolic function (26). In the present study, high PAP with or without respiratory failure was not associated with decreased S'RV, indicating that pulmonary hypertension does not alter systolic right function in newborn infants.The patency of the DA may explain these results in our population. The DA patency prevents supra systemic overload of the right ventricle and a right-to-left shift of the septum; it also avoids life-threatening dysfunction of the right ventricle (27).

The early diastolic trans-tricuspid peak flow velocity (E wave) is proportional to right atrial pressure (28). The E'RV of the RV free wall (E') is a preload-independent marker of RV diastolic relaxation (29). Thus, the E/E’ ratio is considered to be an index of the RV filling pressure. Sciomer et al. (6) showed that a tricuspid E/E' ratio >6 accurately predicts a mean atrial pressure >10 mmHg. The E/E' ratio in our studied population with pulmonary hypertension was > 6, suggesting that elevated PAP is associated with high right atrial pressure in newborn infants. Diastolic RV function in adult patients can be assessed by measuring the IVRT (30, 31). In the present study, the duration of the IVRT of the right ventricle was four times longer in newborn infants with pulmonary hypertension and respiratory failure than in newborn infants with pulmonary hypertension and no respiratory failure. The IVRT has been reported to be prolonged in patients with pulmonary hypertension (30, 31). Previous studies have shown that IVRT increases during the fetal period from 17 weeks of gestation until term (32). It has also been demonstrated that an increase in preload leads to a decrease in IVRT (33). Furthermore, a prior study in newborns weighing <1,250 g reported high IVRT-RV values similar to our PPHN group (34).These consistent findings are in line with the fact that premature newborn present respiratory distress requiring ventilatory support. In addition, other studies showed similar results with increased IVRT in newborn infants with hypoxic-ischemic encephalopathy during therapeutic hypothermia, indicating diastolic dysfunction in this population (35). These data provide evidence for a striking diastolic dysfunction of the right ventricle in newborn infants with PPHN. Diastolic dysfunction may alter RV relaxation. The elevation of end-diastolic right ventricular pressure may increase right-to-left blood shunting through the foramen ovale, worsening hypoxemia. Similar results were obtained in a patient with severe hypoxia with prolongated IVRT and myocardial performance index without modification of late relaxation parameters (35).

The mechanism of PPHN-induced diastolic RV dysfunction is presently unknown. Previous studies have reported that PPHN is associated with increased pro-inflammatory cytokines and oxidative stress (36). Pro-inflammatory cytokines, particularly IL-6, have been shown to impair diastolic ventricular function (37). The newborn heart is more sensitive to inflammation because of its immature muscular arrangement and increased collagen concentration (38).

Previous studies in adults or newborns with congenital diaphragmatic hernias have shown that RV dysfunction may impair LV function (39). It can affect the LV pressure-volume relationship through the interventricular septum, which shifts toward the left cavity and causes the ventricles to compete with each other for space within the pericardium (3, 6, 40). In the present study, no evidence of LV dysfunction was found in newborn infants with pulmonary hypertension. The patency of the DA may explain this discrepancy in our population. Several studies have shown the benefits of maintaining DA patency by augmenting systemic blood flow in the setting of LV failure and reducing the effective afterload on the pressure-loaded right ventricle, thereby alleviating RV dilatation and myocardial dysfunction by acting as a pressure “blow-off” (27, 41).

In our study, we assess pulmonary arterial pressures using the pressure gradient across the patent ductus arteriosus (PDA), a method that has been validated in the literature (17, 18). However, it is important to acknowledge that this assessment is influenced by systemic arterial pressure variations and may introduce a potential bias in the interpretation of pulmonary pressures. To mitigate this potential bias, we specifically included only patients with normal arterial blood pressure at term and excluded those requiring inotropic support or exhibiting hypovolemia. By focusing on patients with stable blood pressure, we aimed to ensure the reliability and validity of the PDA pressure gradient as an estimation of pulmonary arterial pressures in both group.

Our study has several limitations that should be acknowledged. Firstly, the sample size was small, which may limit the generalizability of our findings. Secondly, all measurements were performed by a single observer, introducing the potential for observer bias. It would have been valuable to have a second investigator independently perform the measurements and report the inter-observer variability. Furthermore, the absence of direct hemodynamic data, such as cardiac catheterization, is notable. However, given the invasiveness of this technique, it was not feasible to perform it in our vulnerable population. Instead, we relied on non-invasive measures to assess cardiac function. Another potential limitation is the possibility of selection bias in this small, non-randomized study. Clinical decisions were made by a multidisciplinary team throughout the day to ensure standardized patient care and minimize bias. However, it is important to acknowledge that the possibility of selection bias cannot be completely eliminated. It would have been interesting to include LV IVRT in the TDI analysis of the left ventricle, and this warrants further studies. In summary, while our study provides valuable insights, the limitations regarding sample size, single observer measurements, absence of direct hemodynamic data, and potential selection bias should be taken into consideration when interpreting the results.

## 5. Conclusion

Taken together, we found that, compared with asymptomatic pulmonary hypertension, PPHN is characterized by marked right diastolic ventricular dysfunction, whereas systolic function is preserved. The impaired RV diastolic function causes a decrease in RV compliance. Thus, the hypoxic respiratory failure in PPHN results, at least in part, from the diastolic RV dysfunction-induced increase in right-to-left shunting across the foramen ovale. We propose that the severity of the respiratory failure is more related to the RV diastolic dysfunction than the pulmonary artery pressure.

## Data Availability

The raw data supporting the conclusions of this article will be made available by the authors, without undue reservation.

## References

[1] Storme L Aubry E Rakza T Houeijeh A Debarge V Tourneux P Pathophysiology of persistent pulmonary hypertension of the newborn: impact of the perinatal environment. Arch Cardiovasc Dis. (2013) 106:169–77. 10.1016/j.acvd.2012.12.005 23582679

[2] Levin DL Heymann MA Kitterman JA Gregory GA Phibbs RH Rudolph AM. Persistent pulmonary hypertension of the newborn infant. J Pediatr. (1976) 89:626–30. 10.1016/S0022-3476(76)80405-2 784932

[3] Jain A McNamara PJ. Persistent pulmonary hypertension of the newborn: advances in diagnosis and treatment. Semin Fetal Neonatal Med. (2015) 20:262–71. 10.1016/j.siny.2015.03.001 25843770

[4] AbdelMassih AFA Hassan FZ El-Gammal A Tawfik M Nabil D. The overlooked left ventricle in persistent pulmonary hypertension of the newborn. J Matern Fetal Neonatal Med. (2021) 34:72–6. 10.1080/14767058.2019.1598363 30895828

[5] Malowitz JR Forsha DE Smith PB Cotten CM Barker PC Tatum GH. Right ventricular echocardiographic indices predict poor outcomes in infants with persistent pulmonary hypertension of the newborn. Eur Heart J – Cardiovasc Imaging. (2015) 16:1224–31. 10.1093/ehjci/jev071 25851325 PMC4609160

[6] Sciomer S Magrì D Badagliacca R. Non-invasive assessment of pulmonary hypertension: doppler–echocardiography. Pulm Pharmacol Ther. (2007) 20:135–40. 10.1016/j.pupt.2006.03.008 16753319

[7] Koestenberger M Sallmon H Avian A Cantinotti M Gamillscheg A Kurath-Koller S Ventricular-ventricular interaction variables correlate with surrogate variables of clinical outcome in children with pulmonary hypertension. Pulm Circ. (2019) 9:2045894019854074. 10.1177/2045894019854074 31099302 PMC6542130

[8] Garcia MJ Thomas JD Klein AL. New Doppler echocardiographic applications for the study of diastolic function. J Am Coll Cardiol. (1998) 32:865–75. 10.1016/S0735-1097(98)00345-3 9768704

[9] Brodin L-A. Tissue Doppler, a fundamental tool for parametric imaging. Clin Physiol Funct Imaging. (2004) 24:147–55. 10.1111/j.1475-097X.2004.00542.x 15165284

[10] Nestaas E Schubert U de Boode WP El-Khuffash A. Tissue Doppler velocity imaging and event timings in neonates: a guide to image acquisition, measurement, interpretation, and reference values. Pediatr Res. (2018) 84:18–29. 10.1038/s41390-018-0079-8 30072806 PMC6257218

[11] Friesen RM Schäfer M Burkett DA Cassidy CJ Ivy DD Jone P-N. Right ventricular tissue Doppler myocardial performance Index in children with pulmonary hypertension: relation to invasive hemodynamics. Pediatr Cardiol. (2018) 39:98–104. 10.1007/s00246-017-1733-3 28980052

[12] Peček J Koželj M Lenasi H Fister P. Right ventricular function in neonates during early postnatal period: a prospective observational study. Pediatr Cardiol. (2022) 43:1327–37. 10.1007/s00246-022-02855-7 35229170

[13] Schaefer A Meyer G Hilfikerkleiner D Brand B Drexler H Klein G. Evaluation of tissue Doppler tei index for global left ventricular function in mice after myocardial infarction: comparison with pulsed Doppler tei index. Eur J Echocardiogr. (2005) 6:367–75. 10.1016/j.euje.2005.01.007 16153558

[14] Grignola JC Ginés F Guzzo D. Comparison of the tei index with invasive measurements of right ventricular function. Int J Cardiol. (2006) 113:25–33. 10.1016/j.ijcard.2005.10.012 16325940

[15] Hall RW Anand KJS. Pain management in newborns. Clin Perinatol. (2014) 41:895–924. 10.1016/j.clp.2014.08.010 25459780 PMC4254489

[16] Singh Y Tissot C Fraga MV Yousef N Cortes RG Lopez J International evidence-based guidelines on point of care ultrasound (POCUS) for critically ill neonates and children issued by the POCUS working group of the European society of paediatric and neonatal intensive care (ESPNIC). Crit Care Lond Engl. (2020) 24:65. 10.1186/s13054-020-2787-9 PMC7041196 32093763

[17] Cloez JL Isaaz K Marchal C Morizot D Pernot C. Measurement by Doppler echocardiography of the pulmonary arterial pressure in children with ductus arteriosus. Simultaneous Doppler and hemodynamic study. Arch Mal Coeur Vaiss. (1986) 79:719–24. PMID: 3092772. 3092772

[18] Lu CZ Hu YY Xu QL. Determination of pressure gradient across patent ductus arteriosus with simultaneous continuous wave Doppler and catheterization methods and non-invasive estimation of pulmonary artery pressure. Zhonghua Nei Ke Za Zhi (1991) 30:550–3. PMID: 1806337. 1806337

[19] Aggarwal S Agarwal P Natarajan G. Echocardiographic prediction of severe pulmonary hypertension in neonates undergoing therapeutic hypothermia for hypoxic-ischemic encephalopathy. J Perinatol Off J Calif Perinat Assoc. (2019) 39:1656–62. 10.1038/s41372-019-0442-6 31471580

[20] Aggarwal S Natarajan G. Echocardiographic correlates of persistent pulmonary hypertension of the newborn. Early Hum Dev. (2015) 91:285–9. 10.1016/j.earlhumdev.2015.02.008 25782054

[21] Peterson AL Deatsman S Frommelt MA Mussatto K Frommelt PC. Correlation of echocardiographic markers and therapy in persistent pulmonary hypertension of the newborn. Pediatr Cardiol. (2009) 30:160–5. 10.1007/s00246-008-9303-3 18779989

[22] Price DJA. Tissue Doppler imaging: current and potential clinical applications. Heart. (2000) 84:ii11–8. 10.1136/heart.84.suppl_2.ii11 11040030 PMC1766544

[23] Hori Y Kano T Hoshi F Higuchi S. Relationship between tissue Doppler-derived RV systolic function and invasive hemodynamic measurements. Am J Physiol-Heart Circ Physiol. (2007) 293:H120–5. 10.1152/ajpheart.00097.2007 17322423

[24] Jain A Mohamed A El-Khuffash A Connelly KA Dallaire F Jankov RP A comprehensive echocardiographic protocol for assessing neonatal right ventricular dimensions and function in the transitional period: normative data and Z scores. J Am Soc Echocardiogr. (2014) 27:1293–304. 10.1016/j.echo.2014.08.018 25260435

[25] Eriksen BH Nestaas E Hole T Liestøl K Støylen A Fugelseth D. Myocardial function in premature infants: a longitudinal observational study. BMJ Open. (2013) 3:e002441. 10.1136/bmjopen-2012-002441 23533215 PMC3612763

[26] Patel N Mills JF Cheung MMH. Assessment of right ventricular function using tissue Doppler imaging in infants with pulmonary hypertension. Neonatology. (2009) 96:193–9. 10.1159/000215585 19407463

[27] Le Duc K Mur S Sharma D Aubry E Recher M Rakza T Center for rare disease «congenital diaphragmatic hernia». prostaglandin E1 in infants with congenital diaphragmatic hernia (CDH) and life-threatening pulmonary hypertension. J Pediatr Surg. (2020) 55:1872–8. 10.1016/j.jpedsurg.2020.01.008 32061366

[28] Sadler DB Brown J Nurse H Roberts J. Impact of hemodialysis on left and right ventricular Doppler diastolic filling indices. Am J Med Sci. (1992) 304:83–90. 10.1097/00000441-199208000-00003 1503115

[29] Sundereswaran L Nagueh SF Vardan S Middleton KJ Zoghbi WA Quiñones MA Estimation of left and right ventricular filling pressures after heart transplantation by tissue Doppler imaging. Am J Cardiol. (1998) 82:352–7. 10.1016/S0002-9149(98)00346-4 9708666

[30] Chang S-M Lin C-C Hsiao S-H Lee C-Y Yang S-H Lin S-K Pulmonary hypertension and left heart function: insights from tissue Doppler imaging and myocardial performance Index. Echocardiography. (2007) 24:366–73. 10.1111/j.1540-8175.2007.00405.x 17381645

[31] Melek M Esen O Esen AM Barutcu I Fidan F Onrat E Tissue Doppler evaluation of tricuspid Annulus for estimation of pulmonary artery pressure in patients with COPD. Lung. (2006) 184:121–31. 10.1007/s00408-005-2571-2 16902836

[32] Harada K Rice MJ Shiota T Ishii M McDonald RW Reller MD Gestational age- and growth-related alterations in fetal right and left ventricular diastolic filling patterns. Am J Cardiol. (1997) 79:173–7. 10.1016/s0002-9149(96)00706-0 9193018

[33] Schmitz L Stiller B Koch H Koehne P Lange P. Diastolic left ventricular function in preterm infants with a patent ductus arteriosus: a serial Doppler echocardiography study. Early Hum Dev. (2004) 76:91–100. 10.1016/j.earlhumdev.2003.11.002 14757261

[34] Sirc J Dempsey EM Miletin J. Diastolic ventricular function improves during the first 48 hours-of-life in infants weighting <1250 g. Acta Paediatr Oslo Nor 1992 (2015) 104:e1–6. 10.1111/apa.12788 25163391

[35] Rodriguez MJ Martinez-Orgado J Corredera A Serrano I Arruza L. Diastolic dysfunction in neonates with hypoxic-ischemic encephalopathy during therapeutic hypothermia: a tissue Doppler study. Front Pediatr. (2022) 10:880786. 10.3389/fped.2022.880786 35692972 PMC9174686

[36] Rawat M Lakshminrusimha S Vento M. Pulmonary hypertension and oxidative stress: where is the link? Semin Fetal Neonatal Med. (2022) 27(4):101347. 10.1016/j.siny.2022.101347 35473693 PMC11151383

[37] Prins KW Archer SL Pritzker M Rose L Weir EK Sharma A Interleukin-6 is independently associated with right ventricular function in pulmonary arterial hypertension. J Heart Lung Transplant. (2018) 37:376–84. 10.1016/j.healun.2017.08.011 28893516 PMC5854490

[38] Marijianowski MMH van der Loos CM Mohrschladt MF Becker AE. The neonatal heart has a relatively high content of total collagen and type I collagen, a condition that may explain the less compliant state. J Am Coll Cardiol. (1994) 23:1204–8. 10.1016/0735-1097(94)90612-2 8144790

[39] Patel N Massolo AC Paria A Stenhouse EJ Hunter L Finlay E Early postnatal ventricular dysfunction is associated with disease severity in patients with congenital diaphragmatic hernia. J Pediatr. (2018) 203:400–407.e1. 10.1016/j.jpeds.2018.07.062 30195555

[40] Buckberg GD Hoffman JIE Coghlan HC Nanda NC. Ventricular structure–function relations in health and disease: part I. The normal heart. Eur J Cardiothorac Surg. (2015) 47:587–601. 10.1093/ejcts/ezu278 25086103

[41] Hari Gopal S Patel N Fernandes CJ. Use of prostaglandin E1 in the management of congenital diaphragmatic hernia–A review. Front Pediatr. (2022) 10:911588. 10.3389/fped.2022.911588f 35844758 PMC9283565

